# A Step on the Road to Success

**Published:** 1920-06-19

**Authors:** 


### THE FIGHT AGAINST ANTHRAX.

### A Step on the Road to Success.

Few medical students complete their course without in imagination suffering from at least one serious and fatal disease, so great are the disturbing effects of a little knowledge; and there are few persons who have realised the omnipresence of bacteria who do not first or last suffer from, shall we say, Germiphobia to a greater or less extent. Tubercle bacilli live for months in dust under suitable conditions, garden soil teems with the "vibrion septique" of Pasteur, horse-droppings are full of active tenanus bacilli, and yet we survive!

When we go through a list of our minute vegetable foes, to study how and where they live, and how and where they may be killed, we cannot fail to be struck by the bacillus of anthrax. This organism is indeed a true cosmopolitan; once the dread of French shepherds, its power for ill was greatly reduced by the researches of Pasteur and others, but the disease is still there. England provides a few sporadic cases in animals, as also do all the countries of Europe. The flocks of China seem to be heavily infected, while it is found throughout all Asia. In America it is very fairly prevalent in the South, but is found throughout the whole Continent.

Then as to its powers of living, it seems to be able to grow anywhere, and when its supply of sustenance is exhausted, it forms spores which are more resistant than any other spores known. These spores may be dried for years, heated to the maximum atmospheric temperature for months, frozen for any length of time, and yet, should they reach a suitable animal body they will at once grow, and multiply till this host is killed. Nor have they finished their evil course with the death of their victim, for workers in France were able to show that from the corpses of animals dead of anthrax, worms and movements in the ground could bring virulent organisms up to the surface through six or even twelve feet of earth, and these organisms, ingested by other animals while grazing, will cause that animal too to become infected.

In man the usual manifestation is either as "Malignant Pustule," a local cutaneous infection found in butchers from handling infected meat or animals, or tanners from infected hides, or as "Woolsorters' Disease," a generalised infection resulting from inspiration or ingestion of the organism. Recently there has been much talk of infection by shaving brushes, and this is quite possible, nay, more, it has happened. However, it should not immediately be assumed that every large bacillus found in a shaving-brush is *B. Anthracis*. The examination of cultures from many brushes condemned as infected has shown that the suspected organism was not *B. Anthracis*, but a member of that large group of non-pathogenic organisms known as *B. Mesentericus*.

There have been many attempts to prevent the importation of anthrax. To forbid the importation of hides, wool, or hair from countries where the disease is known is impossible; it would result in stopping the import of these raw materials *in toto*, to the hurt of our own industries and the benefit of our less particular rivals. This has focussed attention on methods of disinfection, for by these alone can workers be protected.

Here, again, the path is beset with difficulties. All the known disinfectants were tried, and when used with sufficient force succeeded in killing both the anthrax bacillus and his spores, but they ruined the material in which these were hidden. Heat to be of any use was enough to bake the wool or hide, and so ruin it. Phenol and other chemicals rotted the material more easily than they killed the bacillus. Sulphuric acid even was tried, and gave results less bad than many seemingly less drastic processes.

The condition of the wool must be first considered. For instance, much that comes from Persia and Mesopotamia is soiled with clotted blood, and in some cases 10 per cent. of the blood clots prove on examination to be infected with anthrax spores. Again, mohair from Turkey, the Cape, or the Van districts often contains blood clots, and these, too, are often infected. Even when no blood-stains or clots can be seen anthrax spores are to be found. These fleeces and skins are also often fouled with excreta and mud. It therefore follows that in addition to their natural protective coat, many of the spores to be destroyed are embedded in very impermeable substances.

As a result of the investigations of the Disinfection Sub-committee of the Departmental Committee on Anthrax, an entirely workable method has been elaborated by which wool and hair can be absolutely sterilised as regards to anthrax, and that without any damage to the material. The wool or hair removed from the hide or skin is, in the ordinary course of events, washed. This wash usually lasts but five to ten minutes, during which time the wool is moved through a shallow trough of soap solution in a series of sharp jerks. In the disinfecting process the time is increased to twenty to thirty minutes, otherwise little or no change is made. The washed wool is then passed through rollers to remove any excess of soap, and then passed into a similar trough containing 2.5 per cent. formaldehyde solution. In this solution the wool remains for twenty minutes or so; it is then again squeezed dry by means of a pair of rollers. It is then slowly transferred to a drying chamber, which it reaches in about ten minutes and in which it remains for about the same time.

Wool so treated, even when it had originally contained clots of blood heavily infected with *B. Anthracis*, was found to be sterile, and when submitted to examination by experts, or during the various processes of spinning and manufacture showed no depreciation in quality. These results were obtained when using an improvised machine, with amateur workers.

A large disinfecting station on this plan is now in course of construction at Liverpool. A site

**The Fight Against Anthrax**—(*continued*).

convenient both for the docks, and also for the large wool warehouses, has been found, and it is hoped shortly to be able to deal with six to eight million pounds of wool per annum. Though this quantity sounds very large, it is less than 10 per cent. of the total quantity of dangerous wool which enters the United Kingdom by way of Liverpool.

This method, admirable though it may be for wool or hair, is not applicable to hides which have to undergo tanning, so that that particular source of anthrax is likely to continue. But now it has been definitely proved that formaldehyde solutions will kill the spores if it can reach them; it should not be a matter of any great difficulty to modify the process so as to meet the needs of the worker in hides. Most of the cases of malignant pustule occur in men whose duties cause them to handle the freshly imported bales of hides. Once tanning is begun the risk of infection goes, but if it were possible to disinfect all hides as they left the ship, even these cases might be avoided.

